# Numerical and Theoretical Analysis of the Inertia Effects and Interfacial Friction in SHPB Test Systems

**DOI:** 10.3390/ma13214809

**Published:** 2020-10-28

**Authors:** Pei Pei, Zhongcai Pei, Zhiyong Tang

**Affiliations:** School of Automation Science and Electrical Engineering, Beihang University, Beijing 100191, China; peipei@buaa.edu.cn (P.P.); peizc@buaa.edu.cn (Z.P.)

**Keywords:** SHPB, inertia effects, interfacial friction, finite element simulation, theoretical analysis

## Abstract

The dynamic properties of materials should be analyzed for the material selection and safety design of robots used in the army and other protective structural applications. Split Hopkinson pressure bars (SHPB) is a widely used system for measuring the dynamic behavior of materials between 10^2^ and 10^4^ s^−1^ strain rates. In order to obtain accurate dynamic parameters of materials, the influences of friction and inertia should be considered in the SHPB tests. In this study, the effects of the friction conditions, specimen shape, and specimen configuration on the SHPB results are numerically investigated for rate-independent material, rate-dependent elastic-plastic material, and rate-dependent visco-elastic material. High-strength steel DP500 and polymethylmethacrylate are the representative materials for the latter two materials. The rate-independent material used the same elastic modulus and hardening modulus as the rate-dependent visco-elastic material but without strain rate effects for comparison. The impact velocities were 3 and 10 m/s. The results show that friction and inertia can produce a significant increase in the flow stress, and their effects are affected by impact velocities. Rate-dependent visco-elasticity material specimen is the most sensitive material to friction and inertia effects among these three materials (rate-independent material, rate-dependent elastic-plastic material, and rate-dependent visco-elastic material). A theoretical analysis based on the conservation of energy is conducted to quantitatively analyze the relationship between the stress measured in the specimen and friction as well as inertia effects. Furthermore, the methods to reduce the influence of friction and inertia effects on the experimental results are further analyzed.

## 1. Introduction

Designers, engineers, and manufacturers are always in search of new and better materials for performance improvements and cost reduction in their robot products to remain competitive in the market. This requirement has resulted in the limited selection of thousands of material choices for designers. Metal, composite material, and polymer are still the main choices for the structural design of robots, especially for their bearing components. In engineering applications, quadruped bionic robots [1], exoskeleton robots [2], and humanoid robots [3] are used in the army and other protective structural applications. Dynamic impact incidents—e.g., the falling of maintenance tools, unintentionally trampling, gravel impact, and hail impact—often occur, which is dangerous to the service safety of robots. Hence, it is essential to investigate the dynamic properties of different materials for a proper selection of materials and robot component safety design.

The split Hopkinson pressure bar (SHPB) is a widely used experimental system to investigate the dynamic compression properties of materials between strain rates of 10^2^ and 10^4^ s^−1^ [4,5]. There are many factors that can influence the accuracy of SHPB experimental results, such as the material response, test conditions and data processing [6,7,8]. Experimental results from SHPB experiments are the stress waves of materials under different strain rates [9,10]. A measurement error of less than 4% dynamic flow stress increment is needed if the strain rate effects are to be reliably characterized, and all sources of error must be examined closely [11,12]. There are two fundamental assumptions in SHPB compression tests, one of which is that the stress and deformation of the specimen is uniform and the other is that there is one-dimensional elastic stress wave propagation in the bars. The accuracy of the experimental results can be ensured by minimizing the influence of factors violating these two assumptions. Various factors have been determined to result in inaccurate SHPB experimental results, among which the inertia effect and interfacial friction are the most critical ones. The axial inertia results in a stress difference between the two ends of the specimen, which violates the first assumption of SHPB experiments. The radial inertia of the specimen during compression leads to multiaxial stress states in the specimen. The stress calculating method based on the second assumption will exceed the true stress of materials. In addition, the interfacial friction generates a radial-directional shear stress on the specimen end surface, which not only changes the stress state in the specimen from uni-axial to multi-axial but also affects the original shape of the specimen cross-section. When the stress waves cross variable cross-section areas, nonuniform deformation is promoted and the stress measurement is affected. Thus, evaluating the influence of inertia effects and interfacial friction in SHPB tests is vital for the accurate determination of the dynamic mechanical properties.

Interfacial friction and inertia effects in SHPB tests have been realized by analytical [13,14,15,16], experimental [17,18,19,20,21,22,23], and numerical approaches [15,16,17,18,19,20,21,22,23,24,25,26,27,28,29]. However, experimental data are susceptible to noise and other sources of error, which cannot be avoided in dynamic tests. Numerical approaches focus only on the existence of inertia effects in SHPB tests. They do not provide a way to quantitative analyze the interfacial friction and inertia effects, nor methods to minimize their influence. Previous analyses have always investigated the effects of interfacial friction together with inertia effects. Meanwhile, few studies have analyzed interfacial friction and inertia effects considering the effect of the tested material properties, which limits the application of these studies.

A theoretical model was firstly established by Davies and Hunter [13]. They found there was additional stress owing to the radial inertia effects in SHPB tests, and derived the actual stress calculation in a cylindrical specimen using the law of energy conservation during impact testing. A slenderness ratio was suggested for a specimen design to eliminate the difference. Based on Davies’ analysis, Samanta [14] took the change rate of the material into consideration, as there will be an acceleration in the specimen during SHPB experiments even if the loading strain rate is constant. Gorham [15] released the axial freedom of specimen ends to the effect of axial and radial inertia effect in SHPB experiments. They also wrote the expression of specimen stress in terms of the mean stress at the two specimen–bar interfaces rather than just the output bar stress, which was more consistent with the experimental results. Sen et al. [16] modified Gorham’s analysis to non-cylindrical specimens with a more complicated expression in strain acceleration terms. The results for non-cylindrical specimens are consistent with those of cylindrical specimens [24].

Various experimental analyses have been conducted by researchers. Jia [17] suggested that the size of the copper specimen should be reduced with the increasing strain rate, which requires a smaller and more precise SHPB experimental system. Casem [18] and Zhang [19] conducted SHPB experiments on polymeric materials with a low density and strength, and concluded that inertial effects have a significant effect on the stress level of polymeric materials in the initial deformation stage, resulting in the fluctuation and peak value of the output signal. Song [20] suggested an annular specimen design to reduce the effect of inertia and proposed an analytical solution for inertia calculation for such a specimen design. Bischoff [21] and Zhang [22] concluded that the inertial confinement is not significant in experiments with a strain rate up to 200 s^−1^. However, it is generally (but not universally) accepted that inertial confinement plays an important role in the SHPB results when the strain rate is higher than 1000 s^−1^; According to experimental observation, Bertholf and Karnes [25] established a two-dimensional numerical model to analyze the effect of inertial and interfacial effects on the material dynamic properties. They concluded that a suitable specimen design and enough lubrication can minimize these factors’ effects. Meng and Li [26] introduced two coefficients to measure the effects of friction and specimen size on the stress uniformity in the specimen when simulating the SHPB experiments by FEM to examine the effects of radial inertia and end friction. Zencker [27] conducted simulations which showed that the optimum specimen slenderness ratio proposed by Davies and Hunter [13] was not accurate as it did not consider the friction effects. Iwamoto and Yokoyama [28] conducted computational simulations to demonstrate the effects of inertia on SHPB measurements considering both rate-dependent and rate-independent material models to demonstrate the inertia effect in SHPB tests.

In the present study, the influence of friction effects and inertia on the dynamic mechanical properties is numerically analyzed, particularly for different materials and loading conditions. An analytical model is established to quantitatively analyze the interfacial friction and inertia effects in SHPB tests. Methods to minimize the influence of interfacial friction and inertia are proposed based on the simulation results and the analytical model.

The structure of the paper is as follows. In Section 2, the specimen deformation characteristics in SHPB experiments are theoretically analyzed. In Section 3, a series of finite element models of SHPB tests are established to study the friction effects and inertia effects. Friction coefficients between 0 and 0.50, the specimen shape (cubic and cylinder), and the specimen slenderness (0.5, 1, 15) are considered. In Section 4, an energy conservation analysis based on the theory by Davies and Hunter (1963) is carried out, and a quantitative equation for the relation of the stress measured in the SHPB to the interfacial friction and inertia effects including the basic material parameters and the experimental conditions is derived. In Section 5, the results of the numerical simulation and analytical model are discussed, based on which methods to minimize the influence of interfacial friction and inertia are proposed.

## 2. Specimen Deformation Characteristic in SHPB

### 2.1. Principle of Split Hopkinson Pressure Bar

**Figure 1:**
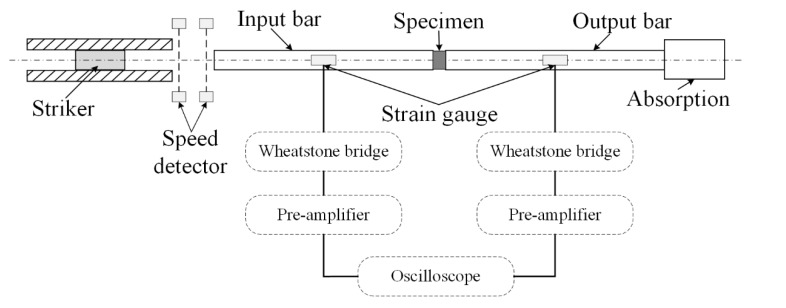
Split Hopkinson Pressure Bar device.

A conventional SHPB experimental device consists of a striker, an incident bar, and a transmit bar, as shown in Figure 1. The specimen is sandwiched between the incident bar and the transmit bar. At the beginning of experiments, the striker impacts the incident bar with an initial velocity $V_{0}$. An incident stress pulse is generated and propagated into the incident bar. When the incident stress pulse reachs the interface between the specimen and bars, a reflected pulse $\sigma_{r}$ is generated in the incident bar and a transmitted pulse $\sigma_{t}$ is generated in the transmitted bar due to the stiffness mismatch between the bars and specimen. These three pulses will be recorded by the strain gauge on the incident bar and transmit bar.

The elastic stress wave propagation speed C, incident pulse $\sigma_{i},$ and the duration of the created incident stress pulse ∆t can be defined as follows:

(1) $$C\;=\;\sqrt{\frac{E}{\rho }},$$

(2) $$\sigma_{i}\;=\;\frac{1}{2}\rho CV_{0},$$

(3) $$∆t\;=\;\frac{2L}{C},$$

where *E* is Young’s modulus of the bar, ρ is the mass density of the bar, $V_{0}$ is the velocity of the striker, and L is the length of the striker.

Based on the assumptions of SHPB, the stress $\sigma_{s}$, strain $\varepsilon_{i},$ and strain rate ${\dot{\varepsilon }}_{s}$ of the specimen can be calculated as follows:

(4) $$\begin{array}{c} \sigma_{s}\;=\;\frac{AE}{2A_{s}}\left(\varepsilon_{i}+\varepsilon_{r}+\varepsilon_{t}\right)\\ \varepsilon_{s}\;=\;\frac{C}{l}\int^{\;}\left(\varepsilon_{i}-\varepsilon_{r}-\varepsilon_{t}\right)dt\\ {\dot{\varepsilon }}_{s}\;=\;\frac{C}{l}\left(\varepsilon_{i}-\varepsilon_{r}-\varepsilon_{t}\right) \end{array}\}\to \varepsilon_{t}\;=\;\varepsilon_{i}+\varepsilon_{r}\to \{\begin{array}{c} \sigma_{s}\;=\;\frac{A}{A_{s}}E\varepsilon_{t}\\ \varepsilon_{s}\;=\;-\frac{2C}{l}\int^{\;}\varepsilon_{r}dt\\ \varepsilon_{t}\;=\;-\frac{2C\varepsilon_{r}}{l} \end{array}.$$

### 2.2. Interfacial Effect in SHPB Tests

**Figure 2:**
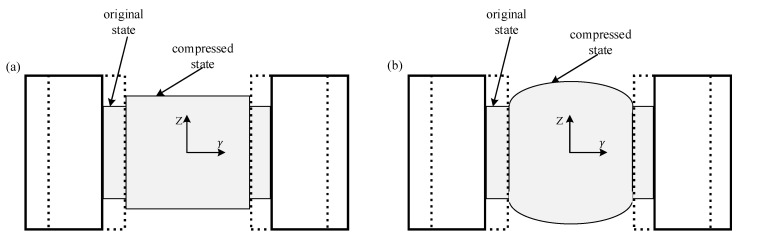
Geometric description of the deformed SHPB specimen under compression. (**a**) Frictionless interface and (**b**) frictional interface.

In a SHPB experiments, for the sufficiently lubricated contact condition the friction coefficient on the interface is zero. The specimen is uniformly shortening in the axial direction (Z direction) and freely expanding in the radial direction (γ direction), as is shown in Figure 2 [30].

For the frictional contact condition, the radial expansion of the specimen ends is a constraint due to the friction forces caused by contact pressure. Consequently, the cross-section area of the specimen varies in the axial direction. For an infinitesimal element along the axial of a deformed specimen, a stress pulse $\sigma_{i}$ propagates from one side to another side, in which the area varies. There will be a stress difference between the two ends due to the stress equivalence requirement in the dynamic tests. The difference is determined by the interfacial coefficient and specimen diameter [28,30,31].

## 3. Finite Element Model of SHPB

A series of nonlinear finite element models using the commercial software ABAQUS/Explicit are established to simulate the effects of interfacial friction and inertia on the dynamic response for different materials and loading conditions. 

**Figure 3:**
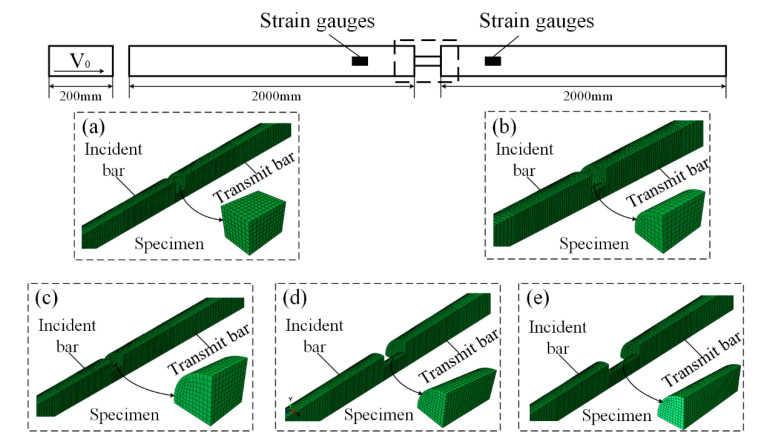
FEA model of a specimen with (**a**) cubic specimen with slenderness 0.66; (**b**) cylinder specimen with slenderness 0.66; (**c**) cylinder specimen with slenderness 0.5; (**d**) cylinder specimen with slenderness 1; (**e**) cylinder specimen with slenderness 1.5.

Since the geometries of bars and specimens are axisymmetric, a quarter of the model was used throughout the simulation work. Both the incident bar and transmit bar are 2000 mm long and modelled with 494,400 C3D8R elements. The striker is 200 mm long with 30,800 C3D8R elements. The dynamic load is applied by imposing the initial velocity on the striker. Two initial impact velocities are considered: 3 and 10 m/s. The stress waves are recorded by the strain gauges as the position in the experiments. Surface-to-surface contact is set between the specimen and bars to prevent interpenetration. Two specimen shapes are considered: a cylinder specimen and a cubic specimen (Figure 3a,b). The friction coefficients are set between 0 and 0.5 with an increment of 0.1. Here, 0 represents ideal lubrication and 0.5 is a large friction coefficient. The range 0–0.5 can cover the possible range of friction coefficients in SHPB tests [30]. To investigate the effect of specimen slenderness on the dynamic response, three specimen slenderness are considered: 0.5, 1, and 1.5 (Figure 3c,e).

The striker and incident/transmitted bars are modelled by linear elastic materials with a Young’s modulus of 150 GPa and Possion’s ratio of 0.3. Specimens are modeled with rate-independent and rate-dependent elastic-plastic material and rate-dependent viscoelasticity material, respectively. 

For rate-independent material, the constituting relationship is described by elastic-plastic materials.

(5) $$\bar{\sigma }\;=\;{\bar{\sigma }}_{0}+A\;{\left({\bar{\varepsilon }}^{p}\right)}^{n},$$

where A and n are the material parameters shown in Table 1.

For rate-dependent elastic-plastic material, the flow stress is described by the Johnson–Cook constitutive model using the experimental results in Refs. [27,28,30,31,32], expressed as follows:

(6) $$\sigma \;=\;\left(A+B{\left({\bar{\varepsilon }}^{pl}\right)}^{n}\right)\left[1+Cln\left({\dot{\bar{\varepsilon }}}^{pl}/{\dot{\bar{\varepsilon }}}_{0}{}^{pl}\right)\right]\left[1-{\left(\frac{T-T_{room}}{T_{melt}-T_{room}}\right)}^{m}\right],$$

where *T* is the temperature parameter; $T_{room}$ is the room temperature and also the reference temperature; $T_{melt}$ is the melt temperature; A, B, C, n, and m are the material parameters and are shown in Table 2.

For rate-dependent viscoelasticity material, the flow stress is described in the Zhu-Wang-Tang (ZWT) constitute model whose parameters are obtained from experimental testing [33] and expressed as follows:

(7) $$\sigma \;=\;E_{0}\varepsilon +\alpha \varepsilon^{2}+\beta \varepsilon^{3}+E_{1}\int_{0}^{t}\dot{\varepsilon }\left(\tau \right)exp\left(-\frac{t-\tau }{\theta_{1}}\right)d\tau +E_{2}\int_{0}^{t}\dot{\varepsilon }\left(\tau \right)exp\left(-\frac{t-\tau }{\theta_{2}}\right)d\tau ,$$

where *E*_0_, *E*_1_, *E*_2_, α, β, $\theta_{1}$, and $\theta_{2}$ are the material constants listed in Table 3. This model is implemented with the explicit dynamic finite element software ABAQUS with the user subroutine to define material behavior (VUMAT).

## 4. Interfacial Effect in SHPB Tests

**Figure 4:**
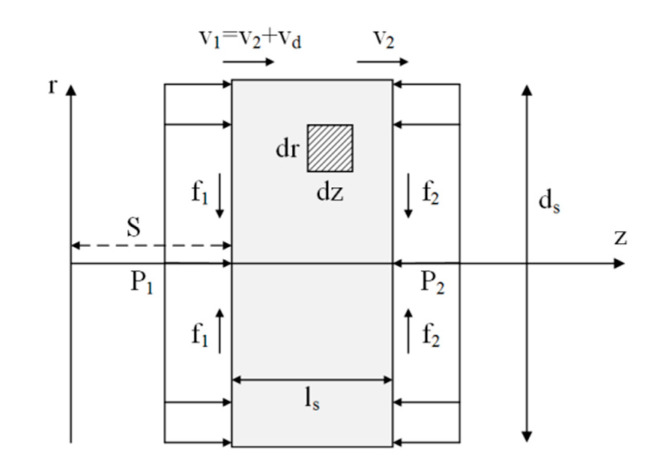
Analysis model of the inertial effect of the specimens in SHPB tests. $d_{s}$ and $l_{s}$ are the diameter and the thickness of a specimen, respectively. S is the reference position in the axial coordinate; $v_{1}$ and $v_{2}$ are the velocities at specimen ends. $v_{d}$ is the deformation velocity of a specimen; P1 and P2 are the pressure forces on the interaction surface between the specimen and bars. $f_{1}$ and $f_{2}$ are the friction forces caused by P1 and P2.

In the SHPB experiments, when the incident stress pulse crosses a variable cross-sectional specimen, the confining effect due to friction will bring a non-uniform stress distribution in the specimen axial direction. The value of the transmitted stress pulse is influenced by the radius difference in the cross section area and the friction coefficient. As the assumption of uniform stress distribution is challenged, the results obtained by the traditional SHPB processing method become unreliable. The analysis model of the inertia effect of the specimen is shown in Figure 4.

The deformation velocity $v_{d}$ of sample can be expressed as:

(8) $$v_{d}\;=\;{\dot{\varepsilon }}_{x}l_{s}.$$

Assuming that the strain rate in the cross-section area of the specimen is uniform, the velocity field of the specimen in the axial direction can be expressed as:

(9) $$v_{z}\left(z\right)\;=\;v_{1}-v_{d}\left(z-S\right)/l_{s}.$$

Assuming that the volume of the specimen is constant during compression, the velocity field of the specimen in radial direction can be expressed as:

(10) $$v_{r}\left(r\right)\;=\;-\mu_{s}v_{d}r/l_{s}.$$

Assuming that the interfacial friction has little influence on the velocity field, temporal change rate of the kinetic energy ${\dot{E}}_{k}$ of the specimen can be expressed as:

(11) $${\dot{E}}_{k}\;=\;\pi \rho_{s}d_{s}{}^{2}\left[\begin{array}{c} \frac{{\dot{d}}_{s}}{d_{s}}\left(\frac{v_{2}{}^{2}l_{s}}{4}+\frac{v_{d}{}^{2}l_{s}}{12}+\frac{v_{2}v_{d}l_{s}}{4}+\frac{\mu_{s}{}^{2}v_{d}{}^{2}d_{s}{}^{2}}{32l_{s}}\right)\\ +\frac{v_{2}{\dot{v}}_{2}l_{2}}{4}+\frac{v_{d}{}^{3}}{24}+\frac{v_{2}{}^{2}v_{d}}{8}+\frac{v_{d}{\dot{v}}_{d}l_{s}}{12}+\frac{v_{2}{\dot{v}}_{d}l_{s}}{8}\\ +\frac{v_{2}v_{d}{}^{2}}{8}+\frac{\mu_{s}{}^{2}}{32}\frac{v_{d}{\dot{v}}_{d}d_{s}{}^{2}}{l_{s}}+\frac{\mu_{s}{}^{2}}{32}\frac{v_{d}{}^{2}d_{s}{\dot{d}}_{s}}{l_{s}}-\frac{\mu_{s}{}^{2}}{64}\frac{v_{d}{}^{3}d_{s}{}^{2}}{l_{s}{}^{2}} \end{array}\right]$$

The temporal change rate of the deformation energy ${\dot{E}}_{p}$ can be expressed as:

(12) $${\dot{E}}_{p}\;=\;\frac{\pi d_{s}{}^{2}}{4}v_{d}E_{s}\varepsilon_{x}.$$

In which *E_s_* is the Young’s modulus of materials.

The temporal rate of change of the external work can be expressed as:

(13) $$\dot{W}=\frac{\pi d_{s}{}^{2}}{4}\left[P_{1}\left(v_{d}+v_{2}\right)-P_{2}v_{2}\right]-\int_{S}\left[f_{1}\left(v_{d}+v_{2}\right)-f_{2}v_{2}\right].$$

Assuming that the friction is uniformly distributed on the end surface, the temporal rate of change of the external work can be expressed as:

(14) $${\dot{W}}_{ext}\;=\;\frac{\pi d_{s}{}^{2}}{4}\left[\sigma_{1}\left(v_{d}+v_{2}-\frac{d_{s}v_{d}\eta }{6l_{s}}\right)-\sigma_{2}\left(v_{2}+\frac{d_{s}v_{d}\eta }{6l_{s}}\right)\right],$$

where η is the friction coefficient.

The equation of motion in the axial direction is:

(15) $$P_{1}-P_{2}\;=\;\rho_{s}l_{s}A_{s}\frac{dv_{s}}{dt},$$

where $v_{s}$ is the rigid body motion velocity of the specimen. Thus,

(16) $$\sigma_{1}-\sigma_{2}\;=\;\rho_{s}l_{s}\left({\dot{v}}_{2}+\frac{{\dot{v}}_{d}}{2}\right).$$

Assuming that the deformation process of the specimen is adiabatic, the conservation of energy can be expressed as:

(17) $${\dot{E}}_{k}+{\dot{E}}_{p}\;=\;{\dot{W}}_{ext}.$$

Substituting Equations (9)–(15) into (16), it can be concluded in Equation (17) if the average stress ($0.5*(P_{1}+P_{2})$) is used to measure the specimen stress in SHPB experiments.

(18) $$\begin{array}{c} \frac{1}{2}\left(\sigma_{1}+\sigma_{2}\right)\left(\frac{d_{s}\eta }{3l_{s}}-1\right)\;=\;\\ E_{s}\varepsilon_{x}+{\ddot{\varepsilon }}_{x}\rho_{s}\left[\frac{\mu_{s}{}^{2}d_{s}{}^{2}}{8}+\frac{l_{s}{}^{2}}{12}\right]+{\dot{\varepsilon }}_{x}{}^{2}\rho_{s}\left[\frac{\mu_{s}{}^{2}d_{s}{}^{2}}{16}-\frac{\mu_{s}{}^{3}d_{s}{}^{2}}{4}+\frac{l_{s}{}^{2}}{4}-\frac{\mu_{s}l_{s}{}^{2}}{3}\right]\\ +v_{1}v_{2}\rho_{s}\left(\frac{1}{2}-\mu_{s}\right) \end{array}.$$

It can be concluded that the strain acceleration as well as the geometrical properties of the specimen are the key factors causing the inertia effects in the SHPB test, so the resulting dynamic strength enhancement can be simply expressed using only the dominant terms, as shown in:

(19) $$\begin{array}{c} \frac{1}{2}\left(\sigma_{1}+\sigma_{2}\right)\left(\frac{d_{s}\eta }{3l_{s}}-1\right)\;=\;\\ E_{s}\varepsilon_{x}+{\ddot{\varepsilon }}_{x}\rho_{s}\left[\frac{\mu_{s}{}^{2}d_{s}{}^{2}}{8}+\frac{l_{s}{}^{2}}{12}\right]+{\dot{\varepsilon }}_{x}{}^{2}\rho_{s}\left[\frac{\mu_{s}{}^{2}d_{s}{}^{2}}{16}-\frac{\mu_{s}{}^{3}d_{s}{}^{2}}{4}+\frac{l_{s}{}^{2}}{4}-\frac{\mu_{s}l_{s}{}^{2}}{3}\right] \end{array}.$$

The interfacial friction can be expressed as:

(20) $$f_{coefficient}\;=\;\frac{d_{s}\eta }{3l_{s}}-1.$$

The inertia effect can be expressed as:

(21) $$\begin{array}{c} ∆\sigma_{inertia}\;=\;\\ {\ddot{\varepsilon }}_{x}\rho_{s}\left[\frac{\mu_{s}{}^{2}d_{s}{}^{2}}{8}+\frac{l_{s}{}^{2}}{12}\right]+{\dot{\varepsilon }}_{x}{}^{2}\rho_{s}\left[\frac{\mu_{s}{}^{2}d_{s}{}^{2}}{16}-\frac{\mu_{s}{}^{3}d_{s}{}^{2}}{4}+\frac{l_{s}{}^{2}}{4}-\frac{\mu_{s}l_{s}{}^{2}}{3}\right]v_{1}v_{2}\rho_{s}\left(\frac{1}{2}-\mu_{s}\right) \end{array}.$$

## 5. Results and Discussion

### 5.1. Influence of Interfacial Friction on SHPB Dynamic Results

**Figure 5:**
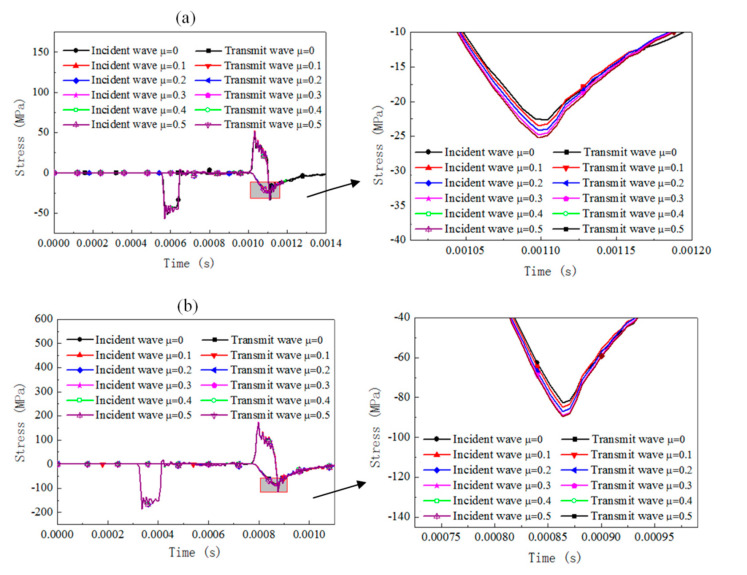
Comparison of the reflected and transmitted waves under different friction conditions and initial impact velocities for rate-independent material (cylinder specimen with slenderness 0.66). (**a**) Impact velocity of 3 m/s, (**b**) impact velocity of 10 m/s.

**Figure 6:**
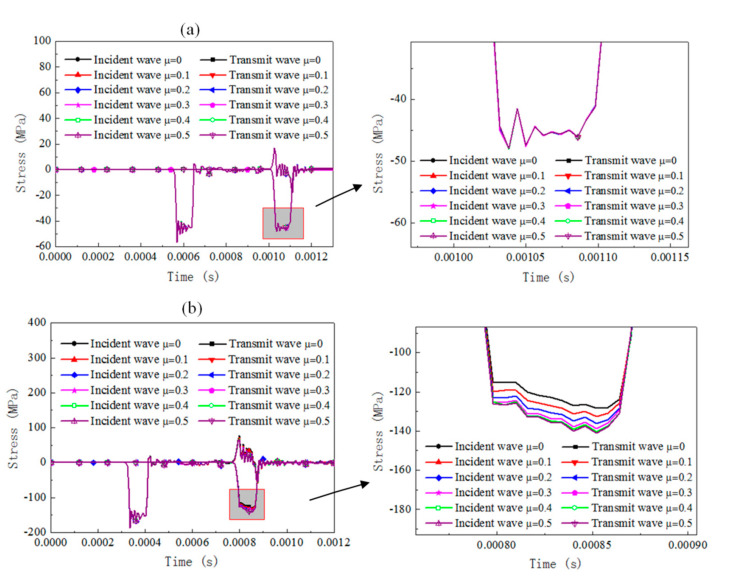
Comparison of the reflected and transmitted waves under different friction conditions and initial impact velocities for rate-dependent elastic-plastic material (cylinder specimen with a slenderness of 0.66). (**a**) Impact velocity of 3 m/s, (**b**) impact velocity of 10 m/s.

**Figure 7:**
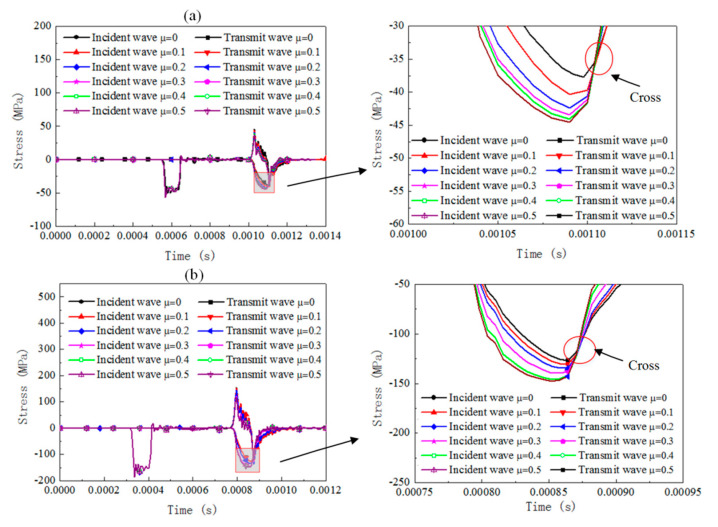
Comparison of the reflected and transmitted waves under different friction conditions and initial impact velocities for the rate-dependent viscoelasticity material (cylinder specimen with a slenderness of 0.66). (**a**) Impact velocity of 3 m/s, (**b**) impact velocity of 10 m/s.

The dynamic response of the cylinder specimen with a slenderness of 0.66 under SHPB compression for different materials is numerically analyzed by considering the friction coefficient set between 0 and 0.5 with an increment 0.1. The results of the stress pulse of the incident bar and transmit bar are presented in Figure 5, Figure 6 and Figure 7).

For rate-independent materials under a 3 m/s impact velocity, the duration of the waves is essentially not affected by the friction coefficient and the transmit stress levels increase by about 13% when the friction coefficient increases from 0 to 0.5. As the impact velocity is 10 m/s, the duration of the waves is slightly affected and the transmit stress pulses increase by about 9%. For rate-dependent elastic plastic materials under a 3 m/s impact velocity, the duration of the waves and the transmit stress levels are affected slightly by the friction coefficient. As the impact velocity increases to 10 m/s, the duration of the waves is still not affected but the transmit stress levels increase by about 10%, with the friction coefficient increasing from 0 to 0.5. For rate-dependent viscoelasticity materials under a 3 m/s impact velocity, the duration of the waves slightly decreases with the friction coefficient increases and the transmit stress levels increase by about 19% when the friction coefficient increases from 0 to 0.5. The growth value of the transmit stress level is about 16% when the impact velocity is 10 m/s. For both the 3 and 10 m/s cases, there is a cross when the viscosity deformation process, after which point the reflected stress level for the case with a smaller friction coefficient was found to be with a higher transmit stress level than the case with a larger friction coefficient.

The interfacial friction brings extra stress and strain rate variation in the SHPB specimen, whose value is related to the testing material properties and impact velocities. Rate-dependent viscoelasticity material is the most sensitive to friction effects among the three materials. However, the increase rate of the transmitted stress value is not influenced by the impact velocity; rate-dependent elastic-plastic material is sensitive to the interfacial friction. The increase rate is significantly influenced by the impact velocity; rate-independent material is influenced by interfacial friction, and its effect is independent of the impact velocity. The conventional SHPB data computed method without consideration of the effect of interfacial friction leads to a falsely higher flow stress for the testing material regardless of the analysis method (three-wave method or two-wave method).

### 5.2. Influence of Inertia on SHPB Dynamic Results

#### 5.2.1. Influence of Specimen Shape on SHPB Dynamic Results

**Figure 8:**
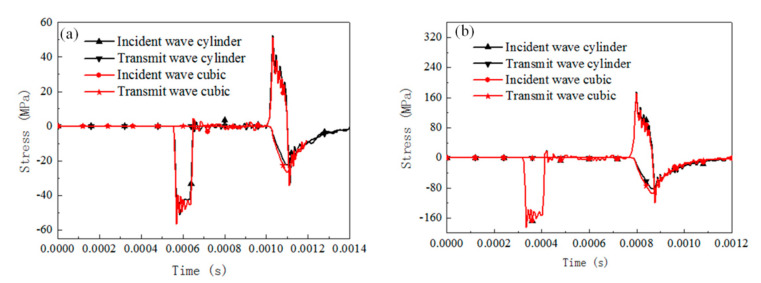
Comparison of the reflected and transmitted waves for cylinder specimen and cubic specimen with rate-independent material (u = 0). (**a**) Impact velocity of 3 m/s, (**b**) impact velocity of 10 m/s.

**Figure 9:**
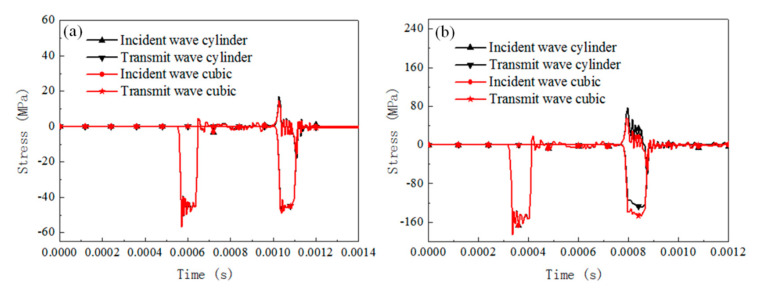
Comparison of the reflected and transmitted waves for cylinder specimen and cubic specimen with rate-dependent elastic-plastic material (u = 0). (**a**) Impact velocity of 3 m/s, (**b**) impact velocity of 10 m/s.

**Figure 10:**
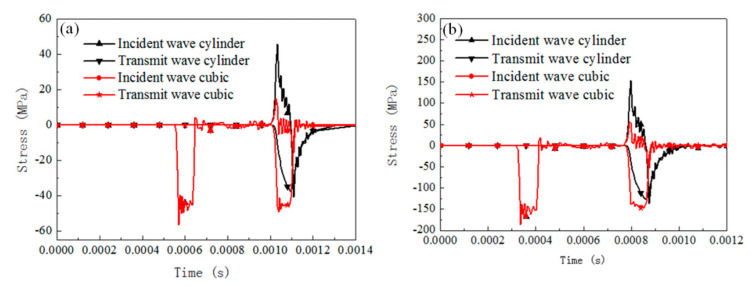
Comparison of the reflected and transmitted waves for cylinder specimen and cubic specimen with rate-dependent viscoelasticity material (u = 0). (**a**) Impact velocity of 3 m/s, (**b**) impact velocity of 10 m/s.

**Figure 11:**
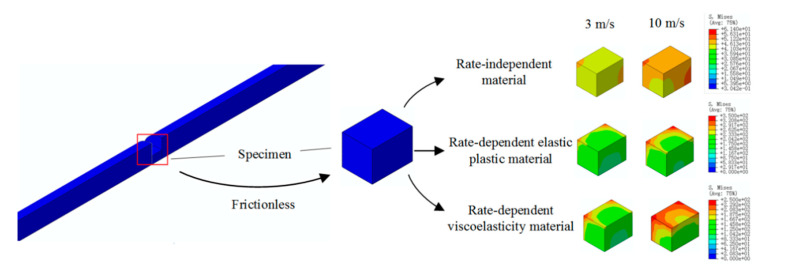
Typical compression deformation of cuboid, equivalent strain distribution at the end of compression.

The dynamic response of specimens with different geometry shapes under SHPB compression for rate-independent material, rate-dependent elastic-plastic material, and rate-dependent viscoelasticity material are numerically analyzed by considering the cubic specimen and cylinder specimen (both specimens with 0.66 slenderness). Two initial impact velocities were considered, 3 and 10 m/s. The contact condition is set frictionless. The results of the stress pulses of the incident and transmitted bars are presented in Figure 8, Figure 9, Figure 10 and Figure 11.

For rate-independent materials, the duration of the waves is not affected significantly by the specimen geometry for both the 3 and 10 m/s impact velocities. When the impact velocity is 3 m/s, the transmit stress level of the cubic specimen is 30% higher than that of the cylinder specimen, and the value decreases to 16% when the impact velocity is 10 m/s. For rate-dependent elastic plastic materials, the duration of the waves is still not affected by the specimen shape for the 3 and 10 m/s impact velocities. When the impact velocity is 3 m/s, the transmit stress level of the cubic specimen is 22.5% higher than that of the cylinder specimen, and the value decreases to 11.7% when the impact velocity is 10 m/s. For rate-dependent viscoelasticity materials, similarly the duration of the waves is not affected by the specimen shape for both impact velocities. When the impact velocity is 3 m/s, the transmit stress level of the cubic specimen is 27% higher than that of the cylinder specimen, and the value decreases to 16% when the impact velocity is 10 m/s. Besides this, the transmit wave shape of the cylinder specimen is a triangle, but it transforms into square wave when the specimen shape is cubic.

Figure 11 shows the stress distribution in the specimen with different materials and impact velocities. For all cases, the stress concentration was located at the corner of the specimen. The rate-dependent viscoelasticity materials show the most severe stress concentration when the impact velocity is 10 m/s.

The specimen geometry has a significant effect on the reflected and transmitted wave stress levels, but not the stress wave duration. Cubic specimens present a higher stress than cylinder specimens regardless of the testing material properties. This is due to the fact that the stress waves would converge at the endpoints of the cuboid specimen and result in a mistaken higher flow stress for testing materials. Rate-dependent viscoelasticity material is the most sensitive to specimen shape among the three materials. However, the increase rate of the transmitted stress value decreases with the increase in the impact velocity. Rate-dependent elastic-plastic material is sensitive to the specimen shape, whose transmitted stress level increases significantly with the impact velocity increasing; rate-independent material is influenced by interfacial friction, and its effect decreases with the impact velocity.

#### 5.2.2. Influence of Specimen Configuration on SHPB Dynamic Results

**Figure 12:**
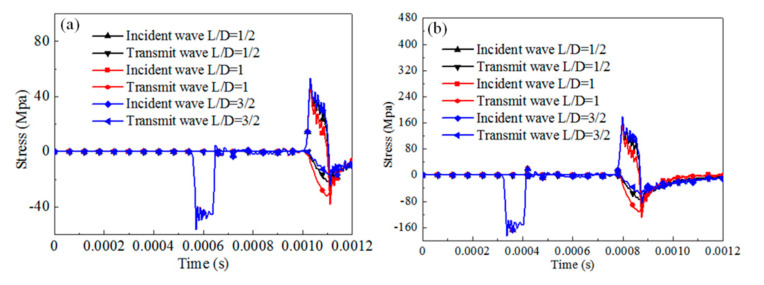
Comparison of the reflected and transmitted waves for different configuration cylinder specimens with rate-independent material (u = 0). (**a**) Impact velocity of 3 m/s, (**b**) impact velocity of 10 m/s.

**Figure 13:**
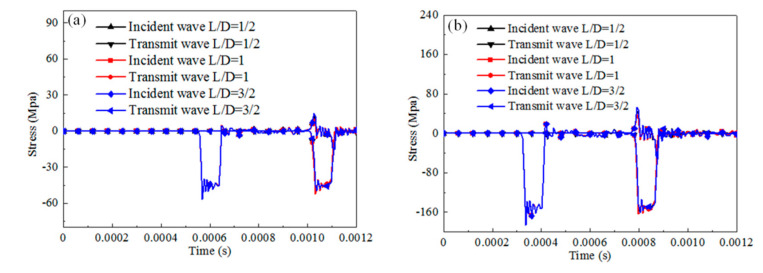
Comparison of the reflected and transmitted waves for different configurations of the cylinder specimen with rate-dependent elastic-plastic material (u = 0). (**a**) Impact velocity of 3 m/s, (**b**) impact velocity of 10 m/s.

**Figure 14:**
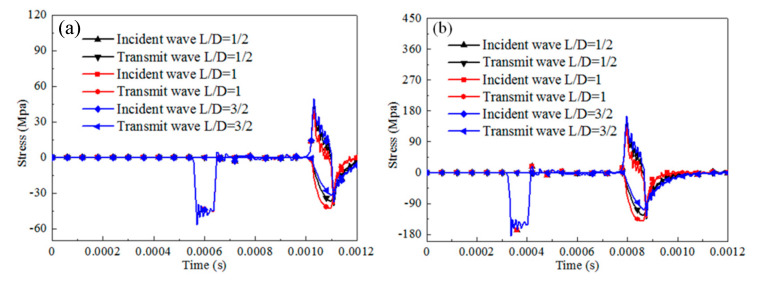
Comparison of the reflected and transmitted waves for different configurations of cylinder specimens with rate-dependent viscoelasticity material (u = 0). (**a**) Impact velocity of 3 m/s, (**b**) impact velocity of 10 m/s.

The dynamic response of the cylinder specimen with different slenderness under SHPB compression for rate-independent material, rate-dependent elastic-plastic material, and rate-dependent viscoelasticity material are numerically analyzed by considering 0.5, 1, 1.5. The contact condition is set frictionless. The results of the stress pulses of the incident and transmitted bars are presented in Figure 12, Figure 13 and Figure 14.

For rate-independent materials, the duration of the waves is not affected by the specimen slenderness regardless of the impact velocities. When the impact velocity is 3 m/s, the transmit stress level of the specimen first increases then decreases with the specimen slenderness increasing. The maximum stress value is 31% higher than the minimum stress value. When the impact velocity is 10 m/s, the transmit stress level shows a similar trend and the ratio of increment is 44%. For rate-dependent elastic plastic materials, the duration of the waves and the transmit stress level are not affected by the specimen slenderness for different impact velocities. For rate-dependent viscoelasticity materials, similar observations as for rate-independent materials are noticed. The duration of the waves is not affected by the specimen slenderness. The transmit stress level of the specimen first increases then decreases with the specimen slenderness increasing. The ratio of increment is 18% for the 3 m/s impact velocity and 17% for the 10 m/s impact velocity.

The specimen geometry has a significant effect on the reflected and transmitted wave stress levels for rate-independent materials and rate-dependent viscoelasticity materials, but not for rate-dependent elastic plastic materials. The transmit stress value first increases then decreases with the increasing specimen slenderness.

### 5.3. Methods to Minimize the Effect of Inertia Effects and End Friction

Thus, ways to minimize the effect of inertia effects and end friction can be considered as follows:

In order to eliminate the effect of term, the coefficient should be approximately zero. Thus, the optimum specimen slenderness ratio should be 1/3 during experiments, which is also the criterion derived by Davies and Hunter in 1963. However, the specimen slenderness ratio increases with the specimen deformation increasing. The criterion derived by Davies and Hunter is only suitable at the experiment’s beginning when the deformation is small. Since it is impossible to ensure that the slenderness ratio stays constant during the experiments, a new criterion which minimizes the absolute sum of the coefficient can be applied to obtain the optimum specimen slenderness ratio. According to the constant strain rate hypothesis and the plastic incompressibility hypothesis, the specimen thickness and diameter can be expressed by its final strain as:

(22) $$l_{s}\;=\;\frac{l_{s0}}{\left(1+\varepsilon \right)},$$

(23) $$d_{s}\;=\;d_{s0}\sqrt{1+\varepsilon }.$$

Thus, the optimum specimen slenderness ratio to minimize the inertia effects can be expressed as:

(24) $$\sum \left[\left(1+\varepsilon \right)\left(\frac{\mu_{s}{}^{2}}{16}-\frac{\mu_{s}{}^{3}}{4}\right)+\left(\frac{1}{4}-\frac{\mu_{s}}{3}\right){\left(\frac{l_{s0}}{d_{s0}}\right)}^{2}\frac{1}{{\left(1+\varepsilon \right)}^{2}}\right]\to 0.$$

**Figure 15:**
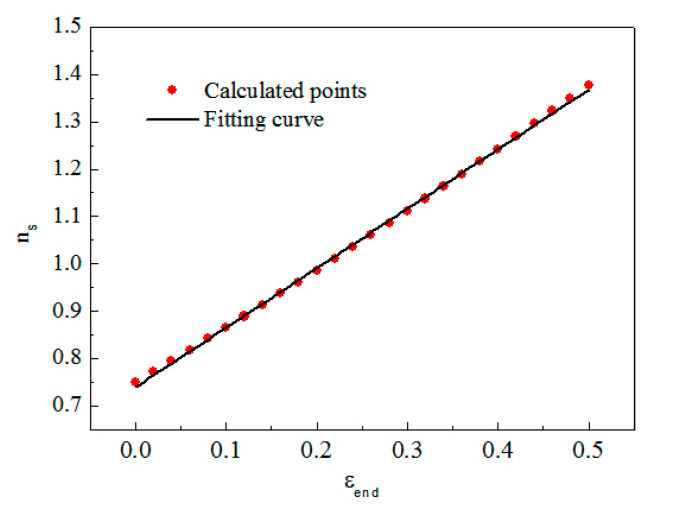
Relation between the optimum specimen slenderness ratio and the final strain of the specimen.

The optimum specimen slenderness ratio is related to its final strain, whose relationship can be fitted by Equation (25), and expressed in Figure 15:

(25) $$n_{s}\;=\;0.73+1.25\varepsilon_{end}.$$

The effect of interfacial friction can be ignored if its influence is less than 5%. Thus, the friction coefficient can be calculated as:

(26) $$\eta <\frac{3l_{s0}}{20d_{s0}\sqrt{\left(1+\varepsilon \right)}}.$$

The above analysis is an efficient method to assess the influence of interfacial friction. A more general method to eliminate the interfacial friction effect is to correct the experimental results according to the theoretical analysis.

## 6. Conclusions

Distinguished with the available literature, friction effects and inertia effects were analyzed theoretically and numerically for different materials in this study. Typical materials were represented by rate-independent material, rate-dependent elastic-plastic material, and rate-dependent visco-elastic material. Inertia effects were investigated by two factors: specimen shape and specimen slenderness. Two impact velocities of 3 and 10m/s were considered. An analytical model was established to quantitatively analyze the interfacial friction and inertia effects in the SHPB tests. Methods to minimize the influence of interfacial friction and inertia were proposed based on the simulation results and analytical model. This study provided material selection suggestions for army and other protective robot designers when they analyze the dynamic properties of materials for a safety design. The main conclusions are as follows:

The interfacial friction brings extra stress for the SHPB specimen, whose values is related to the testing material properties and impact velocities. Rate-dependent viscoelasticity material is the most sensitive to friction effects among the three materials. However, the increase rate of the transmitted stress value is not influenced by the impact velocity; there is a cross in the viscosity deformation process, after which point the reflected stress level for the case with a smaller friction coefficient was found to be with a higher transmit stress level than the case with a larger friction coefficient. The rate-dependent elastic-plastic material is sensitive to the interfacial friction. The increase rate is significantly influenced by the impact velocity; rate-independent material is influenced by interfacial friction, and its effect is independent of the impact velocity.

The specimen geometry has a significant effect on the reflected and transmitted wave stress levels, but not the stress wave duration. Cubic specimens present a higher stress than cylinder specimens regardless of the testing material properties. Rate-dependent viscoelasticity material is the most sensitive to specimen shape among the three materials. However, the increase rate of the transmitted stress value decreases with the impact velocity increasing; rate-dependent elastic-plastic material is sensitive to the specimen shape, whose transmitted stress level increases significantly with the impact velocity increasing. Rate-independent material is influenced by interfacial friction, and its effect decreases with the impact velocity.

The result of impact response of different materials with different interfacial and geometry can be used in the structural design of these materials, especially under impact loads. Under the same loading condition, the difference in the mechanical response of different materials can be attributed to the difference in microstructure. This result can provide some theoretical basis for researchers to improve the material properties.

The specimen geometry has a significant effect on the reflected and transmitted wave stress levels for rate-independent materials and rate-dependent viscoelasticity materials, but not for rate-dependent elastic plastic materials. The transmit stress value first increases then decreases with the increasing specimen slenderness.

The influence of the friction and inertia effect on the SHPB test results is related to the specimen size, deformation strain, strain rate, density of the specimen, and friction coefficient of the interface. Methods to reduce the influence of the inertia effect on the experimental results can be achieved by reducing the influence of these factors, such as using constant strain rate loading experiment technology and a lubricated contact interface. The optimum specimen slenderness ratio is related to its final strain, whose relationship can be described by a linear function. The interface friction is a very complex dynamic process; when the object is deformed in the friction experiments, the process will be even more complex. However, this needs to be researched, since it always occurs in daily life.

## Figures and Tables

**Table 1:** Parameters for the rate-independent material.

*A* (GPa)	*n*
2.95	−10.9

**Table 2:** Parameters for the rate-dependent elastic-plastic material.

*A*	*B*	*n*	*C*	*m*
470	703	0.7	0.019	1.2

**Table 3:** Parameters for the rate-dependent viscoelasticity material.

*T* (°C)	*E*_0_ (GPa)	*A* (GPa)	*B* (GPa)	*E*_1_ (GPa)	*θ*_1_ (s)	*E*_2_ (GPa)	*θ*_2_ (uS)
25	2.95	−10.9	−96.4	0.832	7.33	5.24	40.5

## References

[1] Nelson G., Blankespoor K., Raibert M. (2006). Walking bigdog: Insights and challenges from legged robotics. J. Biomech..

[2] Bogue R. (2009). Exoskeletons and robotic prosthetics: A review of recent developments. Ind. Robot..

[3] Spyros M., Philipp S., Vitchyr P., David C.D. Reactive high-level behavior synthesis for an Atlas humanoid robot. Proceedings of the IEEE ICRA 2016.

[4] Jankowiak T., Rusinek A., Voyiadjis G.Z. (2020). Modeling and design of shpb to characterize brittle materials under compression for high strain rates. Materials.

[5] Chen G., Ren C., Qin X., Li J. (2015). Temperature dependent work hardening in ti-6al-4v alloy over large temperature and strain rate ranges: Experiments and constitutive modeling. Mater. Des..

[6] Brizard D., Ronel S., Jacquelin E. (2017). Estimating measurement uncertainty on stress-strain curves from SHPB. Exp. Mech..

[7] Zhai Y., Li Y., Li Y., Zhang Y., Lu M. (2019). Impact compression test and numerical simulation analysis of concrete after thermal treatment in complex stress state. Materials.

[8] Wang T.T., Shang B. (2014). Three-wave mutual-checking method for data processing of SHPB experiments of concrete. J. Mech..

[9] Wang L.L., Shi S.Q., Chen J.Y., Huang D.J., Shen L.J. (2008). Influences of strain-rate and stress-state on dynamic response of cement mortar. Int. J. Stab. Dyn..

[10] Zou H.R., Yin W.L., Cai C.C., Yang Z., Li Y.B., He X.D. (2019). Numerical investigation on the necessity of a constant strain rate condition according to material’s dynamic response behavior in the SHPB test. Exp. Mech..

[11] Jiang C.L., Cai S.Y., Mao L., Wang Z.C. (2020). Effect of Porosity on Dynamic Mechanical Properties and Impact Response Characteristics of High Aluminum Content PTFE/Al Energetic Materials. Materials.

[12] Chen W., Song B., Frew D.J., Forrestal M.J. (2003). Dynamic small strain measurements of a metal specimen with a Split Hopkinson Pressure Bar. Exp. Mech..

[13] Davies E.D.H., Hunter S.C. (1963). The dynamic compression testing of solids by the method of the Split Hopkinson Pressure Bar. J. Mech. Phys. Solids.

[14] Samanta S.K. (1971). Dynamic deformation of aluminium and copper at elevated temperatures. J. Mech. Phys. Solids.

[15] Gorham D.A., Pope P.H., Field J.E. (1992). An improved method for compressive stress-strain measurements at very high strain rates. Proc. R. Soc. A Math..

[16] Sen O., Tekalur A., Malty P. (2011). On the use of non-cylindrical specimens in a Split-Hopkinson Pressure Bar. J. Strain. Anal. Eng..

[17] Jia D., Ramesh K.T. (2004). A rigorous assessment of the benefits of miniaturization in the Kolsky Bar system. Exp. Mech..

[18] Casem D.T., Dwivedi A.K., Mrozek R.A., Lenhart J.L. (2014). Compression response of a thermoplastic elastomer gel tissue surrogate over a range of strain-rates. Int. J. Solids Struct..

[19] Zhang M., Li Q.M., Huang F.L., Wu H.J., Lu Y.B. (2010). Inertia-induced radial confinement in an elastic tubular specimen subjected to axial strain acceleration. Int. J. Impact Eng..

[20] Song B., Chen W., Ge Y., Weerasooriya T. (2007). Dynamic and quasi-static compressive response of porcine muscle. J. Biomech..

[21] Bischoff P.H., Perry S.H. (1991). Compressive behaviour of concrete at high strain rates. Mater. Struct..

[22] Zhang M., Wu H.J., Li Q.M. (2009). Further investigation on the dynamic compressive strength enhancement of concrete-like materials based on split Hopkinson pressure bar tests. Part I: Experiments. Int. J. Impact Eng..

[23] Chen L., Fang Q., Guo Z., Liu J. (2014). An improved analytical method for restrained RC structures subjected to static and dynamic loads. Int. J. Struct. Stab. Dyn..

[24] Shu D.W., Luo C.Q., Lu G. (2008). X Numerical simulations of the influence of striker bar length on SHPB measurements. Int. J. Mod. Phys. B.

[25] Bertholf L.D., Karnes C.H. (1975). Two-dimensional analysis of the Split Hopkinson Pressure Bar system. J. Mech. Phys. Solids.

[26] Li Q.M., Meng H. (2003). About the dynamic strength enhancement of concrete-like materials in a Split Hopkinson Pressure Bar test. Int. J. Solids Struct..

[27] Zencker U., Clos R. (1999). Limiting conditions for compression testing of flat specimens in the Split Hopkinson Pressure Bar. Exp. Mech..

[28] Iwamoto T., Yokoyama T. (2012). Effects of radial inertia and end friction in specimen geometry in Split Hopkinson Pressure Bar tests: A computational study. Mech. Mater..

[29] Panowicz R., Konarzewski M. (2020). Influence of Imperfect Position of a Striker and Input Bar on Wave Propagation in a Split Hopkinson Pressure Bar (SHPB) Setup with a Pulse-Shape Technique. Appl. Sci..

[30] Zhong W.Z., Rusinek A., Jankowiak T., Abed F., Bernier R., Sutter G. (2015). Influence of interfacial friction and specimen configuration in Split Hopkinson Pressure Bar system. Tribol. Int..

[31] Liu P., Han X., Hu D., Jiang C. (2017). Sensitivity and uncertainty analysis of SHPB tests for concrete materials. Int. J. Appl. Mech..

[32] Qin J., Chen R., Wen X., Lin Y., Liang M., Lu F. (2013). Mechanical behaviour of dual-phase high-strength steel under high strain rate tensile loading. Mater. Sci. Eng. A.

[33] Shi S., Gan S., Wang L. (1990). The thermo-viscoelastic mechanical behavior of an aeronautical PMMA under impact loading. J. Ningbo Univ..

